# National surveillance of *Neisseria gonorrhoeae* antimicrobial susceptibility and epidemiological data of gonorrhoea patients across Brazil, 2018–20

**DOI:** 10.1093/jacamr/dlac076

**Published:** 2022-07-05

**Authors:** Hanalydia de Melo Machado, Jéssica Motta Martins, Marcos André Schörner, Pamela Cristina Gaspar, Alisson Bigolin, Mauro Cunha Ramos, Willian Antunes Ferreira, Gerson Fernando Mendes Pereira, Angélica Espinosa Miranda, Magnus Unemo, Maria Luiza Bazzo, Brazilian-GASP Network, Simone Veloso Faria de Carvalho, Maria Rita Rabelo Costa, Luciane Guimarães Dias, Elly Rodrigo Porto, Lidiane da Fonseca Andrade, Glaura Regina de Castro e Caldo Lima, Viviane Furlan Lozano, Maria Luiza Bazzo, Felipe de Rocco, Fernando Hartmann Barazzetti, Guilherme Kerber, Hanalydia de Melo Machado, Jéssica Motta Martins, Ketlyn Buss, Mara Cristina Scheffer, Marcos André Schörner, Ronaldo Zonta, Mauro Cunha Ramos, Maria Rita Castilhos Nicola, Maria Cristina Cecconi, Barbara Suely Souza de Noronha, Cleiby Andrade dos Santos, Francinete Motta Lopes, Jairo de Souza Gomes, Jamile Izan Lopes Palhesta Júnior; Paulo Tadeu Cavalcante Saif, Willian Antunes Ferreira, Miralba Freire, André Ramos, Felipe Nogueira M. Carvalho, Aida Politano, Roberto José Carvalho da Silva, Sandra de Araújo; Claudio Campos do Porto, Roberta Alessandra Lima Bocalon, Ursula de Oliveira Machado de Souza, Rafael Mialski, Keite da Silva Nogueira, Mônica Baumgardt Bay, Manoella do Monte Alves, Juliana Cintra Campos, Luíz Fernando Aires Junior, Larissa de Oliveira Camargo, Lis Aparecida de Souza Neves, Ana Paula Luchetta Paes, Felipe Barufaldi, Henrique Dib Oliveira Reis, Luiz Sérgio D’Oliveira Rocha, Marta Inês Cazentini Ribeiro, Paulo da Silva, Fabiana Rezende Amaral, François José de Figueiroa, Anesia Maria Siqueira Barbosa, Ana Albertina Araujo, Maria Goretti Varejão, Fernanda Garnier de França Mendes, Valdelucia Oliveira Cavalcanti, Paulo Gabriel Lima Ribeiro, Bruno Ishigami, Lucas Caheté, Cássia Maria Zoccoli

**Affiliations:** Molecular Biology, Microbiology and Serology Laboratory (LBMMS), Federal University of Santa Catarina, Florianópolis, Brazil; Molecular Biology, Microbiology and Serology Laboratory (LBMMS), Federal University of Santa Catarina, Florianópolis, Brazil; Molecular Biology, Microbiology and Serology Laboratory (LBMMS), Federal University of Santa Catarina, Florianópolis, Brazil; Department of Chronic Diseases and STI, Brazilian Ministry of Health, Brasília, Brazil; Public Health Postgraduate Program, Brasilia University, Brasilia, Brazil; Department of Chronic Diseases and STI, Brazilian Ministry of Health, Brasília, Brazil; Brazilian STD Society, Porto Alegre, Brazil; Alfredo da Mata Foundation, Manaus, Brazil; Department of Chronic Diseases and STI, Brazilian Ministry of Health, Brasília, Brazil; Department of Chronic Diseases and STI, Brazilian Ministry of Health, Brasília, Brazil; WHO Collaborating Centre for Gonorrhoea and Other STIs, Department of Laboratory Medicine, Microbiology, Faculty of Medicine and Health, Örebro University, Örebro, Sweden; Institute for Global Health, University College London (UCL), London, UK; Molecular Biology, Microbiology and Serology Laboratory (LBMMS), Federal University of Santa Catarina, Florianópolis, Brazil

## Abstract

**Objectives:**

To (i) describe the nationwide antimicrobial susceptibility of *Neisseria gonorrhoeae* (NG) isolates cultured across Brazil in 2018–20 and compare it with NG antimicrobial resistance data from 2015–16, and (ii) present epidemiological data of the corresponding gonorrhoea patients in 2018–20.

**Methods:**

Twelve representative sentinel sites cultured NG isolates from men with urethral discharge. Susceptibility to eight antimicrobials was examined using agar dilution method, according to WHO standards. The consenting participants were invited to provide epidemiological data.

**Results:**

In total, 633 NG isolates (one isolate per participant) were analysed, and 449 (70.9%) questionnaires were answered. Heterosexual (68.2%) and homosexual (23.1%) sexual orientations were common, and most prevalent types of unprotected sexual intercourse were vaginal insertive (69.9%), oral giving (56.6%) and anal insertive (47.4%). The levels of *in vitro* NG resistance to ciprofloxacin, tetracycline, benzylpenicillin, azithromycin, cefixime, gentamicin, spectinomycin and ceftriaxone were 67.3%, 40.0%, 25.7%, 10.6%, 0.3%, 0%, 0% and 0%, respectively. Compliance with the recommended first-line ceftriaxone 500 mg plus azithromycin 1 g therapy was high (90.9%).

**Conclusions:**

Compared with 2015–16, ciprofloxacin resistance has remained high and azithromycin and cefixime resistance rates have increased in Brazil. Resistance remained lacking to ceftriaxone, gentamicin and spectinomycin, which all are gonorrhoea treatment options. The increasing azithromycin resistance in Brazil and internationally may threaten the future use of azithromycin in dual regimens for treatment of gonorrhoea. Consequently, continued and enhanced quality-assured surveillance of gonococcal AMR, and ideally also treatment failures and including WGS, is imperative in Brazil and worldwide.

## Introduction

Gonorrhoea is a common sexually transmitted infection (STI), caused by *Neisseria gonorrhoeae* (gonococcus), with an estimated global incidence of 82.4 million cases among adults in 2020.^1^ Gonorrhoea can result in serious complications and sequelae, disproportionally affecting women, including pelvic inflammatory disease, ectopic pregnancy, infertility and increased HIV transmission. Effective, accessible and affordable antimicrobial treatment in conjunction with conventional prevention, rapid diagnosis and epidemiological measures are the mainstays for management and control of gonorrhoea.^2,3^

It is a grave concern that *N. gonorrhoeae* has developed or acquired resistance to all antimicrobials introduced for treatment since the 1930s. Over the past 70–80 years, treatment options have diminished rapidly due to the emergence and spread of antimicrobial resistance (AMR) to all drugs previously used or considered for first-line treatment (sulphonamides, penicillins, tetracyclines, spectinomycin, early-generation cephalosporins, trimethoprim combinations, macrolides and fluoroquinolones).^3–6^ In most global settings, the third-generation, extended-spectrum cephalosporins (ESCs) ceftriaxone (injectable) and cefixime (oral) are the only remaining options for first-line empirical antimicrobial monotherapy of gonorrhoea. However, in the past two decades, gonococcal strains with *in vitro* and clinical resistance or decreased susceptibility to ceftriaxone and cefixime have also emerged globally.^3–12^ The emergence and international spread of MDR and sporadic XDR gonococcal strains and treatment failures with ESCs have evolved gonorrhoea into a great public health issue, alerting about the future prospect of untreatable gonorrhoea.^3–9^ Accordingly, enhanced global AMR surveillance is imperative.^7–9^

The surveillance of *N. gonorrhoeae* AMR has in most South American countries been limited, sporadic, lacking representativeness and epidemiological data, and even completely absent in many countries.^7–9^ In Brazil, syndromic management of STIs was implemented in 1993,^13^ which resulted in limited aetiological diagnosis of STIs including gonococcal culture and subsequent antimicrobial susceptibility testing. However, in recent years aetiological diagnosis of STIs has been increasingly reestablished and, in 2015, the first national gonococcal AMR surveillance programme was established.^11^ The first round of AMR surveillance identified, for example, high levels of ciprofloxacin resistance and directly informed revisions of the national treatment guidelines, i.e. in 2017 ciprofloxacin was replaced with ceftriaxone (500 mg) in combination with azithromycin (1 g) for first-line empirical therapy for uncomplicated gonococcal infections.^14^ However, it was also concluded that the AMR surveillance should be enhanced and include additional representative isolates (increased number of isolates and from additional geographic settings), additional antimicrobials and epidemiological data of the patients.

The present study aimed to (i) describe the antimicrobial susceptibility of *N. gonorrhoeae* isolates cultured across Brazil in 2018–20 and compare it with *N. gonorrhoeae* AMR data from 2015–16,^11^ and (ii) present epidemiological data of the corresponding male patients with gonorrhoea. These types of data are crucial to inform the national STI and gonorrhoea treatment guidelines^14^ and national STI surveillance programmes in Brazil. Improvements compared with the previously published first national gonococcal AMR surveillance in Brazil^11^ included: five additional sentinel surveillance sites were included; the number of examined gonococcal isolates was increased; two additional antimicrobials were examined; the latest panel of WHO *N. gonorrhoeae* reference strains was used for quality control; and epidemiological data were collected.

## Materials and methods

### Sentinel sites, biological samples and patient epidemiological data

Twelve sentinel sites appropriately representing all the five main Brazilian regions were selected for patient recruitment and subsequent sample collection. All 12 sites were visited for provision of clinical and laboratory training, standardization and quality assurance of sampling procedures. Subsequently, consecutive men aged ≥18 years with urethral discharge were, after written informed consent, enrolled from August 2018 to December 2020. Exclusion criteria were as follows: individuals that had (i) not had their sexual debut; (ii) been exposed to a suspected sexual abuse; (iii) been receiving systemic antimicrobial therapy ≤7 days prior to attendance and/or (iv) been using topical medications in the urogenital region. All participants were invited to fill in an epidemiological questionnaire, which included queries regarding sexual orientation, gender identity, type of unprotected sexual intercourse and treatment received for current gonorrhoea episode (Table 1). All patients were aimed to be treated empirically in accordance with the national treatment guidelines.^14^ From each participant, a urethral sample was collected using a urethral swab, which was placed in Amies transport medium (Copan, Brescia, Italy).

**Table 1.** Epidemiological and treatment information for gonorrhoea patients (*n* = 449) across Brazil 2018–20

Epidemiological data	Percentage
Sexual orientation	
Heterosexual	68.2
Homosexual	23.1
Bisexual	7.8
Not reported	0.9
Gender identity	
Male cis	87.1
Female trans	0.6
Transvestite	0.2
Not reported	12.0
Unprotected sexual intercourse	
Anal insertive	47.4
Anal receptive	14.3
Vaginal insertive	69.9
Oral giving	56.6
Oral receptive	49.2
Therapy	
CRO 500 mg IM + AZM 1 g PO	90.9
CIP 500 mg PO + AZM 1 g PO	4.0
Other scheme	1.8
Not reported	3.3

CRO, ceftriaxone; IM, intramuscular; AZM, azithromycin; PO, per os/orally; CIP, ciprofloxacin.

#### N. gonorrhoeae culture

The urethral swab samples were inoculated on non-selective chocolate agar (Laborclin, Pinhais, Brazil) and selective Thayer–Martin medium (Laborclin). The agar plates were incubated at 35 ± 1°C in a humid 5% CO_2_-enriched atmosphere for 24 h. If no growth was observed after 24 h, the agar plates were incubated for additional 24 h. Suspected *N. gonorrhoeae* colonies were preserved in tryptic soy broth supplemented with 20% glycerol at −80°C before shipment to the reference laboratory, i.e. Molecular Biology, Microbiology and Serology Laboratory (LBMMS), Federal University of Santa Catarina, Florianópolis. At LBMMS, colonies were species-identified as *N. gonorrhoeae* using Gram stain, catalase and oxidase tests, VITEK^®^2 system (bioMérieux, Marcy-l'Étoile, France), and a duplex in-house PCR targeting the *porA* pseudogene and 16S rRNA gene.^15,16^

### Antimicrobial susceptibility testing

The MICs (mg/L) of ceftriaxone, cefixime, azithromycin, ciprofloxacin, spectinomycin, gentamicin, benzylpenicillin and tetracycline were determined by the agar dilution method, in accordance with recommendations by CLSI.^17^ For quality control of each MIC determination, five gonococcal reference strains were selected from CLSI (ATCC 49226) and WHO (WHO F, G, K, L, M, N, O, P, U, V, W, X, Y, Z^18^). An agreement of ± 1 MIC log_2_ dilution between the measured MIC and the reference strain MIC was required. Etest strips (bioMérieux) were used to confirm high MICs of ceftriaxone, cefixime and azithromycin. The MICs were interpreted using clinical breakpoints recommended by EUCAST, where available.^19^ The clinical breakpoints (susceptible, resistant) were as follows: ceftriaxone and cefixime (≤0.125 mg/L, >0.125 mg/L); ciprofloxacin (≤0.03 mg/L, >0.06 mg/L); benzylpenicillin (≤0.06 mg/L, >1.0 mg/L); tetracycline (≤0.5 mg/L, >2.0 mg/L); and spectinomycin (≤64 mg/L, >64 mg/L).^19^ For azithromycin and gentamicin, for which EUCAST and CLSI do not state any clinical interpretative criteria, the epidemiological cut-off (ECOFF; MIC >1.0 mg/L,^19^ referred to as resistant hereafter) and previously published breakpoints (≤4 mg/L, >16 mg/L), respectively, were applied.^20^

### Ethics approval

The study was approved by the Committee of Ethics in Research with Human Beings, Brazil; assent number 2.524.656 and CAAE 83053818.4.0000.0121.

## Results

### N. gonorrhoeae isolates

In total, 1189 urethral discharge samples (one sample per patient) were collected, and 838 suspected gonococcal isolates were cultured and subsequently sent frozen to the reference laboratory LBMMS. However, especially due to suboptimal storage in three sentinel sites, only 633 viable species-verified *N. gonorrhoeae* isolates were available for antimicrobial susceptibility testing. Of these isolates (*n *= 633), 34.9% (*n* = 221) were cultured in the Southeast region [Belo Horizonte (*n* = 98), Ribeirão Preto (*n* = 94), São Paulo (*n* = 15), São José dos Campos (*n* = 14)]; 22.4% (*n* = 142) in South [Florianópolis (*n* = 72), Porto Alegre (*n* = 57), Curitiba (*n* = 13)]; 16.9% (*n* = 107) in Northeast [Recife (*n* = 97), Salvador (*n* = 10)]; 15.3% (*n* = 97) in North (Manaus); and 10.4% (*n* = 66) in Central-West (Distrito Federal). The geographic distribution and number of *N. gonorrhoeae* isolates obtained from each sentinel site across Brazil is illustrated in Figure S1 (available as Supplementary data at *JAC-AMR* Online).

### Epidemiological data of gonorrhoea patients

Out of the 633 included patients with gonorrhoea, 449 (70.9%) responded to the epidemiological questionnaire. These responses are summarized in Table 1. Briefly, the mean age of respondents was 28.1 years (median age: 26 years), ranging from 18 to 70 years. Most prevalent were brown people (47.2%), followed by Caucasians (30.7%) and black people (20.1%).

Most of the respondents self-declared as heterosexuals (68.2%) and male cis (87.1%), although 23.1% and 7.8% declared themselves as homosexual or bisexual, respectively. Type of unprotected sexual intercourse was a multiple-choice question. Vaginal insertive intercourse was the most frequently reported practice (69.9%), followed by oral giving (56.6%). Insertive anal sex (47.4%) and receptive oral sex (49.2%) were also common (Table 1).

In total, 90.9% of respondents received the currently recommended dual therapy (ceftriaxone plus azithromycin),^14^ 4.0% a dual therapy based on ciprofloxacin plus azithromycin, and 1.8% other regimens, including azithromycin 1 g (*n* = 4), ceftriaxone 1 g, ceftriaxone 500 mg plus doxycycline, ceftriaxone 500 mg plus metronidazole, or doxycycline (one respondent each) (Table 1).

### Antimicrobial susceptibility and resistance

The results of the antimicrobial susceptibility testing of all gonococcal isolates (*n* = 633) are summarized in Table 2. On a national level, 67.3% and 40.0% of isolates were resistant to ciprofloxacin and tetracycline, respectively. The vast majority of isolates (97.0%) showed resistance (25.7%) or susceptibility, increased exposure (71.3%) to benzylpenicillin. In total, 10.6% and 0.3% (*n* = 2; from Belo Horizonte and Ribeirão Preto in Southeast region, respectively) of isolates were resistant to azithromycin and cefixime, respectively. No isolates were resistant to gentamicin, although 31.3% showed susceptibility, increased exposure. All isolates were susceptible to ceftriaxone and spectinomycin.

**Table 2.** Antimicrobial susceptibility of *N. gonorrhoeae* isolates (*n* = 633) collected across Brazil in 2018–20, by Brazilian region

Antimicrobial	Brazilian region					Total (*n* = 633)
	North (*n* = 97)	Northeast (*n* = 107)	Central-West (*n* = 66)	Southeast (*n* = 221)	South (*n* = 142)	
Ceftriaxone, %						
Susceptible (MIC ≤0.125 mg/L)^19^	100	100	100	100	100	100
Resistant (MIC >0.125 mg/L)^19^	0	0	0	0	0	0
Cefixime, %						
Susceptible (MIC ≤0.125 mg/L)^19^	100	100	100	99.1	100	99.7
Resistant (MIC >0.125 mg/L)^19^	0	0	0	0.9	0	0.3
Azithromycin, %						
Susceptible (MIC ≤1 mg/L)^19^	96.9	94.4	92.4	86.4	83.8	89.4
Resistant (MIC >1 mg/L)^19^	3.1	5.6	7.6	13.6	16.2	10.6
Ciprofloxacin, %						
Susceptible (MIC ≤0.03 mg/L)^19^	23.7	39.3	34.8	24.4	40.8	31.6
Susceptible, increased exposure^19^	1.0	3.7	0	0.9	0	1.1
Resistant (MIC > 0.06 mg/L)^19^	75.3	57.0	65.2	74.7	59.2	67.3
Spectinomycin, %						
Susceptible (MIC ≤64 mg/L)^19^	100.0	100.0	100.0	100.0	100.0	100.0
Resistant (MIC >64 mg/L)^19^	0	0	0	0	0	0
Gentamicin, %						
Susceptible (MIC ≤4 mg/L)^20^	70.1	71.0	69.7	73.3	58.5	68.7
Susceptible, increased exposure^20^	29.9	29.0	30.3	26.7	41.5	31.3
Resistant (MIC >16 mg/L)^20^	0	0	0	0	0	0
Benzylpenicillin, %						
Susceptible (MIC ≤0.06 mg/L)^19^	7.2	2.8	3.0	0.5	4.2	3.0
Susceptible, increased exposure^19^	63.9	71.0	78.8	66.5	80.3	71.3
Resistant (MIC >1 mg/L)^19^	28.9	26.2	18.2	33.0	15.5	25.7
Tetracycline, %						
Susceptible (MIC ≤0.5 mg/L)^19^	35.1	56.1	48.5	46.6	52.8	48.0
Susceptible, increased exposure^19^	11.3	6.5	18.2	11.8	14.1	12.0
Resistant (MIC >2 mg/L)^19^	53.6	37.4	33.3	41.6	33.1	40.0

North (Alfredo da Mata Tropical Dermatology and Venereology Foundation, Manaus, Amazonas); Northeast (Specialized State Center in Diagnosis, Care and Research, Salvador, Bahia; AIDS Health Foundation and Central Laboratory, Recife, Pernambuco; Giselda Trigueiro Hospital and Federal University of Rio Grande do Norte, Natal, Rio Grande do Norte); Central-West (Asa Sul Polyclinic, Brasília, Distrito Federal); Southeast (STI/AIDS Reference and Training Center, São Paulo, São Paulo; Belo Horizonte Municipal Health Secretariat, Belo Horizonte, Minas Gerais; Reference Center for Infectious Diseases, São José dos Campos; Adolfo Lutz Institute and Ribeirão Preto Municipal Health Secretariat, Ribeirão Preto, São Paulo); and South (Curitiba Municipal Health Secretariat and Clinic Hospital Complex of Federal University of Paraná, Curitiba, Paraná; Clinical Analysis Department, University Hospital, Federal University of Santa Catarina; Florianópolis Municipal Health Secretariat, Florianópolis, Santa Catarina; and Sanitary Dermatology Outpatient Clinic, Porto Alegre, Rio Grande do Sul).

**Figure 1.**
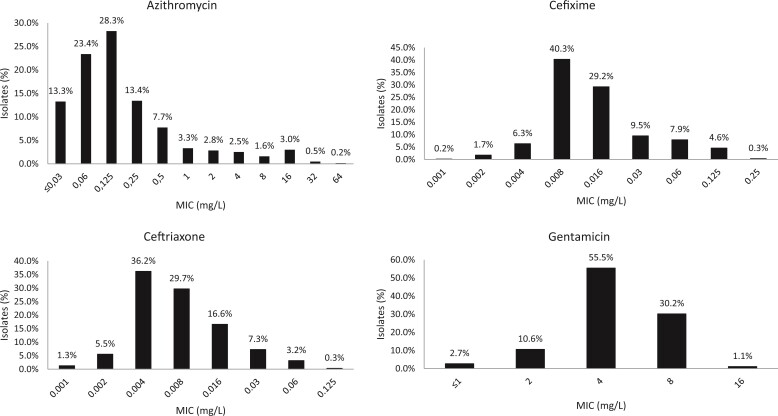
MIC distributions for azithromycin, cefixime, ceftriaxone and gentamicin in *N. gonorrhoeae* isolates collected across Brazil in 2018–20.

In Figure 1, the MIC distributions for azithromycin, ceftriaxone, cefixime and gentamicin are presented.

The MIC distribution for ceftriaxone showed that 0.3% (2/633) of isolates had a ceftriaxone MIC of 0.125 mg/L, which is at the clinical ceftriaxone resistance breakpoint stated by EUCAST (MIC >0.125 mg/L).^19^ The percentage of isolates with low ceftriaxone MICs (≤0.016 mg/L) was high (89.3%). The two cefixime-resistant isolates had cefixime MICs of 0.25 mg/L and, in addition, 4.6% (*n* = 29) of isolates had a cefixime MIC of 0.125 mg/L, i.e. at the clinical cefixime resistance breakpoint.^19^ The majority of isolates (78.4%) had a low azithromycin MIC of ≤0.25 mg/L, however, 5.3% of isolates showed a low level of azithromycin resistance (MICs of 2–4 mg/L) and 5.3% a higher level of azithromycin resistance (MICs of 8–64 mg/L) (Figure 1).

Concerning resistance to multiple antimicrobials, 8.7% (*n* = 55) of the isolates were resistant to both azithromycin and ciprofloxacin and 1.6% (*n* = 10) of isolates were resistant to four antimicrobials (azithromycin, ciprofloxacin, tetracycline and benzylpenicillin).

Notably, the resistance levels to azithromycin (10.1% versus 5.8%) and ciprofloxacin (70.6% versus 67.6%) were higher among heterosexual respondents compared with MSM (i.e. homosexual plus bisexual respondents).

## Discussion

This study presents the nationwide AMR surveillance of *N. gonorrhoeae* isolates cultured across Brazil in 2018–20, quality assured in accordance with WHO standards and controls,^18^ and relevant epidemiological data of the corresponding male patients with gonorrhoea. Improvements compared with the previously published first national gonococcal AMR surveillance in Brazil (2015–16)^11^ included that 12 geographically representative sentinel sites were surveyed (five new sites to improve national representativeness); the number of examined gonococcal isolates was increased (by 15.1% despite three sentinel sites not managing to maintain the viability of many isolates and the COVID-19 pandemic occurring during the last 9 months of surveillance, which consumed healthcare, staff, testing and resources); the latest panel of WHO 2016 *N. gonorrhoeae* reference strains^18^ was used for quality control; two additional antimicrobials (spectinomycin and gentamicin) were examined; and epidemiological data were collected, including e.g. sexual orientation, gender identity, type of unprotected sexual intercourse and treatment received.

As in 2015–16,^11^ high rates of resistance to ciprofloxacin, tetracycline and benzylpenicillin were observed in all the Brazilian regions. Even after excluding ciprofloxacin from the recommended treatment of urethral discharge syndrome and uncomplicated gonococcal infections in 2017,^14^ resistance to ciprofloxacin continued to increase, i.e. in 2018–20 it exceeded 74% in the Southeast and North regions, and it was ≥57% in all regions. This is in accordance with ciprofloxacin resistance data from most other countries in South America and the entire WHO Region of the Americas.^7–9^ However, a low (1.1%) ciprofloxacin resistance rate was recently published from Jamaica.^21^ As observed in the WHO Global Gonococcal Antimicrobial Surveillance Programme (WHO GASP), ciprofloxacin resistance rates are high globally.^7–9^

Azithromycin resistance rates in Brazil increased from 6.9% (recalculated using the current EUCAST ECOFF)^19^ in 2015–16^11^ to 10.9% in 2018–20, with 16.2% resistance in the South region, including the city Florianopolis where >30% of isolates were resistant to azithromycin. Notably, resistance to azithromycin was >5% (5.6%–16.2%) in all regions, except in the North region (3.1%). However, no isolates with high-level resistance to azithromycin (MIC ≥256 mg/L) were found. Such isolates have been sporadically identified in many countries internationally,^6–10^ including in neighbouring Argentina where the azithromycin resistance rate also has increased in the recent years.^22^ The increasing azithromycin resistance rate in Brazil and internationally^9^ is a major concern, which may threaten the use of ceftriaxone plus azithromycin dual therapies in Brazil and internationally,^14,23–26^ and continuous, quality-assured global surveillance of azithromycin resistance is imperative. In Brazil, if gonococcal azithromycin resistance is proven, based on enhanced quality-assured AMR surveillance, to remain high or increasing across the country an exclusion of azithromycin from the ceftriaxone plus azithromycin dual therapy needs to be considered. However, in countries such as Brazil where most gonorrhoea cases are treated based on syndromic management, due to the limited aetiological gonorrhoea diagnosis, an exclusion of azithromycin requires additional considerations that also involve other STIs such as *Chlamydia trachomatis* and *Mycoplasma genitalium* infections. Furthermore, the AMR surveillance in Brazil is still relatively limited and the spread of sporadic ceftriaxone-resistant strains cannot be excluded. Because test of cure is not used in Brazil and concomitant resistance to azithromycin and ceftriaxone in gonococcal strains remains rare, azithromycin in the dual therapy may cover the treatment of these rare ceftriaxone-resistant gonorrhoea cases. Finally, a clinical resistance breakpoint for azithromycin would be very valuable, i.e. it remains unclear how high azithromycin MICs must be to cause treatment failure using azithromycin monotherapy, especially as it is recommended in a dual therapy together with ceftriaxone. Notable, the present study overlapped for approximately 9 months in 2020 with the COVID-19 pandemic, which as in most other countries negatively affected the recruitment of patients with gonorrhoea at sexual and reproductive health services.^27–29^ Furthermore, it cannot be excluded that the overuse of azithromycin during the COVID-19 pandemic in Brazil and many other countries has impacted the azithromycin resistance rates in gonococci (as well as many other bacterial species).

Regarding the oral ESC cefixime, two (0.3%) resistant isolates (MIC = 0.25 mg/L) were identified, and both were cultured in the Southeast region. In the previous national AMR surveillance in Brazil from 2015–16,^11^ only one cefixime-resistant isolate (0.2%) was found, i.e. in Brasilia (Central-West region). The low level of cefixime resistance is promising. However, 29 (4.6%) additional isolates were bordering cefixime resistance, i.e. had a MIC of 0.125 mg/L,^19^ and *N. gonorrhoeae* isolates with decreased susceptibility and resistance to ESCs have significantly increased in the neighbouring Argentina.^30^ Notably, cefixime is not used in Brazil, which likely reduces the selection pressure for ESC resistance in Brazil. Accordingly, the resistance and decreased susceptibility to cefixime in Brazil is likely due to importation of gonococcal strains with decreased ESC susceptibility or selection by the use of other cephalosporins or penicillins. A WGS study of Brazilian gonococcal isolates from 2018–20 is under planning, i.e. to elucidate the AMR determinants causing the phenotypic resistance (with emphasis on ESCs and azithromycin), to further understand the dynamics of the gonococcal population in Brazil, and to compare Brazilian strains with international gonococcal strains.

No resistance to the injectable ESC ceftriaxone or the aminocyclitol spectinomycin was found in Brazil in 2018–20. This is exceedingly promising as ceftriaxone is the last remaining option for empirical first-line monotherapy for gonorrhoea treatment and spectinomycin is included in the recommended treatment regimens when first-line therapy fails in many international gonorrhoea treatment guidelines.^23,24,31–33^ Nevertheless, due to concerns regarding spectinomycin resistance development and the low spectinomycin cure rates for pharyngeal gonococcal infections,^3,6–9,24,34^ spectinomycin should not be used in monotherapy if pharyngeal gonorrhoea has not been appropriately excluded. No resistance to gentamicin was either found among gonococcal isolates across Brazil in 2018–20. Nevertheless, 31.3% of isolates showed a decreased susceptibility (susceptibility, increased exposure) and also gentamicin-susceptible gonococcal isolates can cause treatment failure with gentamicin 240 mg plus doxycycline, as recently shown in Malawi where gentamicin has been the recommended first-line treatment for uncomplicated gonorrhoea in several decades.^35^ The 2020 Brazilian treatment guideline recommends ceftriaxone 500 mg plus azithromycin 1 g as first-line empirical treatment for syndromic management and for aetiologically diagnosed gonorrhoea,^14^ and the present study confirms a high adherence to this treatment regimen (90.9%). Worryingly, ciprofloxacin 500 mg plus azithromycin 1 g and other mostly suboptimal treatment regimens were given to 4.0% and 1.8% of responding participants, respectively. Notably, in the Brazilian treatment guideline,^14^ gentamicin and spectinomycin are treatment options for gonococcal infections when the recommended first-line therapy has failed.

The potential limitations of the present study included that no gonococcal isolates were collected from women or extragenital sites such as the rectum and pharynx. The importance of including isolates also from extragenital sites was further strengthened by the fact that unprotected oral and anal sex were reported by >50% of respondents. Particularly the pharynx has been stated as an anatomical site where gonococcal strains can persist without resulting in symptoms, where AMR can emerge due to acquisition of AMR determinants from co-existing non-gonococcal *Neisseria* species, and the pharyngeal infections are substantially more difficult to cure.^3,5–9,24^ Furthermore, the coverage of reporting on the epidemiological variables was suboptimal, i.e. 70.9% of participants filled in the epidemiological questionnaire. Finally, the loss of approximately 200 viable isolates due to suboptimal storage, which has been addressed for future surveillance rounds, may potentially have slightly biased the AMR rates in some few sentinel sites, however, we do not consider that this issue substantially biased any national AMR rates or main conclusions of the present study.

In conclusion, compared with the first national gonococcal AMR surveillance in Brazil (2015–16),^11^ the resistance to ciprofloxacin has remained high, and the resistance rates for azithromycin and cefixime have increased. However, resistance remained lacking to ceftriaxone, gentamicin and spectinomycin, which all are gonorrhoea treatment options. In Brazil, the compliance to the recommended first-line empirical dual therapy of ceftriaxone 500 mg plus azithromycin 1 g^14^ was high (90.9%). Nevertheless, the increasing azithromycin resistance in Brazil and internationally^9^ may threaten the future use of azithromycin in gonorrhoea dual-therapy regimens. Consequently, continued and enhanced quality-assured surveillance of gonococcal AMR, and ideally also treatment failures at a minimum at some sentinel sites and including WGS, is imperative in Brazil and worldwide. Collection of epidemiological data of patients with gonorrhoea in Brazil also showed that the resistance rates for azithromycin and ciprofloxacin were higher among heterosexual men compared with MSM (homosexual plus bisexual men), and data on sexual practice provided evidence that extragenital sites should also be tested and included in the gonococcal AMR surveillance. The gonococcal AMR surveillance in Brazil will be further improved in the coming few years by inclusion of Brazil in the WHO/CDC Enhanced GASP (EGASP),^36,37^ which provides standardized and quality-assured AMR data in conjunction with epidemiological and clinical data. In the future, the WHO/CDC EGASP^36,37^ will also include a WGS component, for genomic epidemiology and AMR prediction, and test of cure, where feasible. Ultimately, effective, affordable and accessible new antimicrobials and/or gonococcal vaccine(s) will be required.

## Supplementary Material

dlac076_Supplementary_Data

## Members of the Brazilian-GASP Network

**Belo Horizonte:** Simone Veloso Faria de Carvalho, Maria Rita Rabelo Costa, Luciane Guimarães Dias; **Brasília:** Elly Rodrigo Porto, Lidiane da Fonseca Andrade, Glaura Regina de Castro e Caldo Lima, Viviane Furlan Lozano; **Florianópolis:** Maria Luiza Bazzo, Felipe de Rocco, Fernando Hartmann Barazzetti, Guilherme Kerber, Hanalydia de Melo Machado, Jéssica Motta Martins, Ketlyn Buss, Mara Cristina Scheffer, Marcos André Schörner, Ronaldo Zonta; **Porto Alegre:** Mauro Cunha Ramos, Maria Rita Castilhos Nicola, Maria Cristina Cecconi; **Manaus:** Barbara Suely Souza de Noronha; Cleiby Andrade dos Santos; Francinete Motta Lopes; Jairo de Souza Gomes; Jamile Izan Lopes Palhesta Júnior; Paulo Tadeu Cavalcante Saif; Willian Antunes Ferreira; **Salvador:** Miralba Freire, André Ramos, Felipe Nogueira M. Carvalho, Aida Politano; **São Paulo:** Roberto José Carvalho da Silva; Sandra de Araújo; Claudio Campos do Porto; Roberta Alessandra Lima Bocalon; Ursula de Oliveira Machado de Souza; **Curitiba:** Rafael Mialski, Keite da Silva Nogueira; **Natal:** Mônica Baumgardt Bay, Manoella do Monte Alves; **São José dos Campos:** Juliana Cintra Campos, Luíz Fernando Aires Junior, Larissa de Oliveira Camargo; **Ribeirão Preto:** Lis Aparecida de Souza Neves, Ana Paula Luchetta Paes, Felipe Barufaldi, Henrique Dib Oliveira Reis, Luiz Sérgio D’Oliveira Rocha, Marta Inês Cazentini Ribeiro, Paulo da Silva, Fabiana Rezende Amaral; **Recife:** François José de Figueiroa, Anesia Maria Siqueira Barbosa, Ana Albertina Araujo, Maria Goretti Varejão, Fernanda Garnier de França Mendes, Valdelucia Oliveira Cavalcanti, Paulo Gabriel Lima Ribeiro, Bruno Ishigami, Lucas Caheté; **Laboratório Santa Luzia:** Cássia Maria Zoccoli

## Supplementary data

Figures S1 is available as Supplementary data at *JAC-AMR* Online.
