# Supplementary material for: National surveillance of Neisseria gonorrhoeae antimicrobial susceptibility and epidemiological data of gonorrhoea patients across Brazil, 2018–20
Source: JAC Antimicrob Resist. 2022 Jul 5;4(4):dlac076. doi: 10.1093/jacamr/dlac076 (PMC9252985; doi:10.1093/jacamr/dlac076)
Supplement: dlac076_Supplementary_Data [file dlac076_supplementary_data.docx]

**Supplementary data**


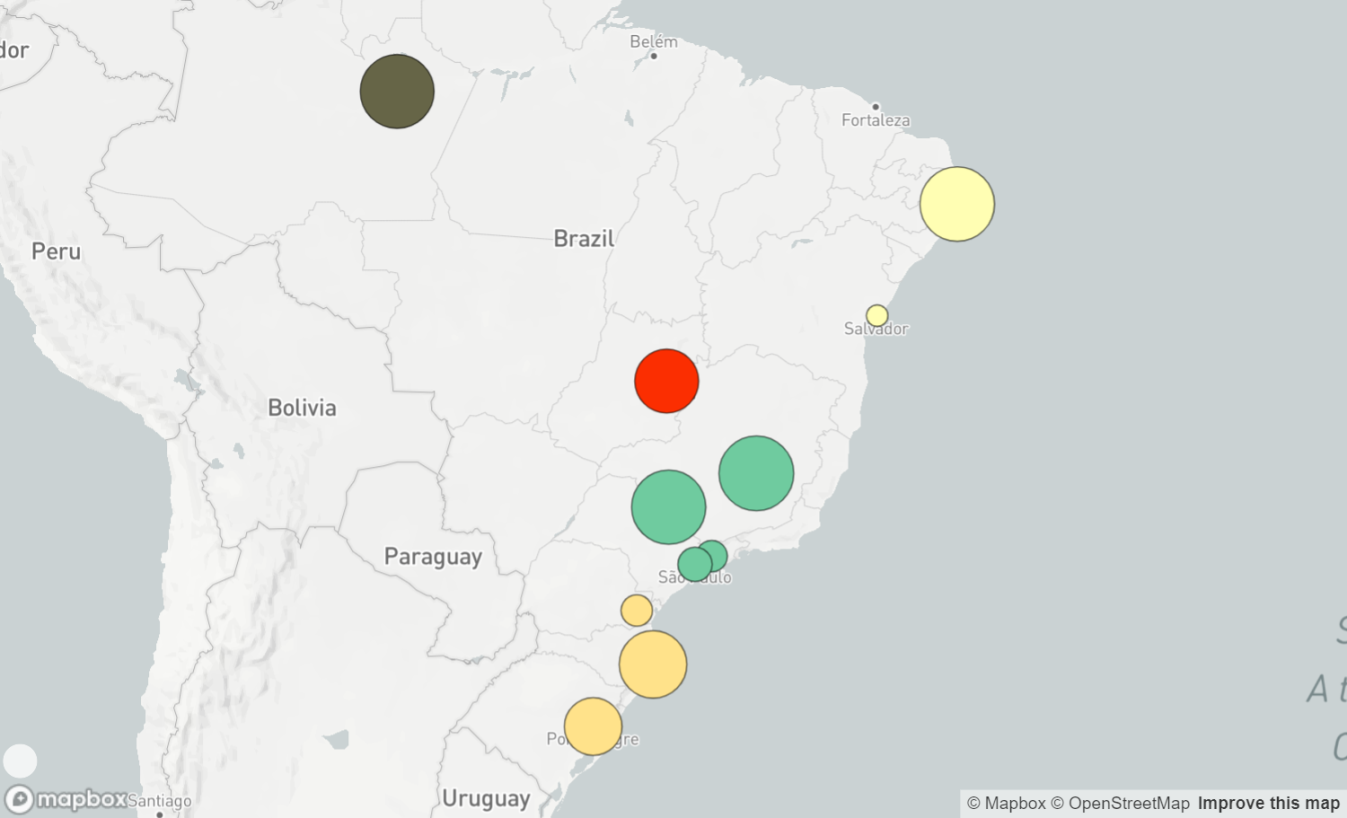


**Figure S1.** Distribution of examined *Neisseria gonorrhoeae* isolates across Brazil in 2018–20. The size of each circle corresponds to the number of isolates in each sentinel site.
